# Contralateral prophylactic mastectomy rate and predictive factors among patients with breast cancer who underwent multigene panel testing for hereditary cancer

**DOI:** 10.1002/cam4.1519

**Published:** 2018-05-07

**Authors:** Nisreen Elsayegh, Rachel D. Webster, Angelica M. Gutierrez Barrera, Heather Lin, Henry M. Kuerer, Jennifer K. Litton, Isabelle Bedrosian, Banu K. Arun

**Affiliations:** ^1^ Department of Breast Medical Oncology The University of Texas MD Anderson Cancer Center Houston Texas; ^2^ Department of Clinical Cancer Genetics The University of Texas MD Anderson Cancer Center Houston Texas; ^3^ Department of Biostatistics The University of Texas MD Anderson Cancer Center Houston Texas; ^4^ Department of Surgical Oncology The University of Texas MD Anderson Cancer Center Houston Texas

**Keywords:** *BRCA* carriers, CPM, multigene panel, non‐*BRCA* carriers, VUS

## Abstract

Although multigene panel testing is increasingly common in patients with cancer, the relationship between its use among breast cancer patients with non‐*BRCA* mutations or variants of uncertain significance (VUS) and disease management decisions has not been well described. This study evaluated the rate and predictive factors of CPM patients who underwent multigene panel testing. Three hundred and fourteen patients with breast cancer who underwent multigene panel testing between 2014 and 2017 were included in the analysis. Of the 314 patients, 70 elected CPM. Election of CPM by gene status was as follows: *BRCA* carriers (42.3%), non‐*BRCA* carriers (30.1%), and VUS (10.6%). CPM election rates did not differ between non‐*BRCA* carriers and *BRCA* carriers (*P* = 0.6205). Among non‐*BRCA* carriers, negative hormone receptor status was associated with CPM (*P* = 0.0115). For those with a VUS, hormone receptor status was not associated with CPM (*P* = 0.1879). Although the rate of CPM between *BRCA* carriers and non‐*BRCA* carriers was not significantly different, the predictors of CPM were different in each group. Our analyses shed the light on the increasing use of CPM among patients who are non‐*BRCA* carriers as well those with a VUS. Our study elucidates the differing predictive factors of CPM election among *BRCA* carriers, non‐*BRCA* carries, and those with a VUS. Our findings reveal the need for providers to be cognizant that non‐*BRCA* genes and VUS drive women to elect CPM despite the lack of data for contralateral breast cancer risk associated with these genes.

## Introduction

Although most breast cancers are sporadic, approximately 5–10% of breast cancers are hereditary, caused by a germline mutation in a myriad of genes implicated in carcinogenesis 1, 2, 3. Mutations in the *BRCA1* and *BRCA2* genes account for 60–80% of inherited breast cancers 4. Defining mutation carrier status is crucial because carriers have a 43–84% risk of developing breast cancer and up to a 65% risk of developing contralateral breast cancer (CBC) 4, 5. Rarer, highly penetrant genes, such as *CDH1*,* PTEN*,* STK11*, and *TP53*, have similarly been shown to be associated with significantly increased risks of developing breast cancer and some potential association with an increased risk of developing CBC 6, 7, 8, 9.

In the past decade, moderately penetrant genes associated with breast cancer, such as *ATM*,* CHEK2*, and *PALB2*, have also been shown to increase the risk of developing breast cancer (between 18.3% and 44%) 10, 11. Studies on these more recently described genes have yet to definitively determine the likelihood of second primary breast cancers owing to the paucity of data. Multiple sources report a significant increase in CBC risk among *CHEK2* mutation carriers, yet other studies did not show a significant association between *CHEK2* and CBC 12, 13, 14, 15. The WECARE study showed that *ATM* mutations are associated with an elevated risk of CBC, but that association is largely dependent on the use of radiotherapy during treatment of initial breast cancer 16, 17, 18. Tischkowitz et al. also published data from the WECARE study showing a higher rate of *PALB2* mutations in women with CBC compared with those with unilateral breast cancer 19. Many of these studies had small sample sizes or presented conflicting data, suggesting that the risk of developing CBC in patients with moderately penetrant gene mutations is still up for debate.

Clinical guidelines developed over the past two decades, such as those presented by the National Comprehensive Cancer Network (NCCN), recommend additional breast cancer screening and consideration of contralateral prophylactic mastectomy (CPM) for breast cancer patients with *BRCA1* and *BRCA2* mutations to address the risk of developing a second primary cancer. The most recent iteration of these guidelines now addresses the appropriate screening considerations for individuals with deleterious mutations in moderately penetrant genes, like the guidelines for rarer, highly penetrant genes with a less established association with risk of developing a second primary breast cancer. The National Comprehensive Cancer Network (NCCN) guidelines encourage consideration of family history for CPM decisions. In the absence of sufficient data and clear consensus guidelines, patients and their physicians are tasked with determining the appropriate course of treatment as the rate of CPM increases among all patients with breast cancer 20, 21.

While there has been an uptake in multigene panel testing in patients with cancer, the management trends of breast cancer patients with non‐BRCA mutations or variants of uncertain significance (VUS) have not been well described. This study aimed to evaluate the rate and predictive factors of CPM in a cohort of individuals who underwent multigene panel testing. We also aimed to determine whether predictors of CPM differed by gene implicated or result type.

## Methods

Three hundred and fifty patients diagnosed with ductal carcinoma in situ or invasive breast cancer who underwent genetic counseling and multigene panel testing between 2014 and 2017 were identified from a prospectively maintained research registry in the Department of Clinical Cancer Genetics at The University of Texas MD Anderson Cancer. The study was approved by the MD Anderson institutional review board. Each patient underwent genetic testing and received pretest genetic counseling per standard of care. The following genes were included in the multigene panel: *APC (2)*,* ATM (17)*,* BARD1 (2)*,* BRCA1* & *BRCA2 (71)*,* BRIP1 (4)*,* CDH1 (5)*,* CDKN2A (2)*, CHEK2 *(19), MEN1 (1),* MLH1 *(1)*,* MUTYH (2)*,* NBN (1)*,* PALB2 (17)*,* PMS2 (2)*,* PTEN (5)*,* RAD51C (1)*, and *TP53 (11)*. After the testing, all results were disclosed by a genetic counselor.

Patients with bilateral breast cancer and patients who had CPM before they underwent multigene panel testing were excluded from our analysis, as were patients with a negative genetic test result. After excluding the 36 patients who elected CPM before genetic testing, we were left with 314 patients who had a pathogenic or likely pathogenic mutation or VUS. For analysis simplicity and the very small number of non‐*BRCA* genes included in the panel, we combined all the non‐*BRCA* carriers together, all patients with VUS in either *BRCA* or non‐*BRCA* genes together. Therefore, the analysis compared three groups: non‐*BRCA* carriers (all other genes listed above), *BRCA* (*BRCA1* or *BRCA2*) carriers, and those with a VUS.

We examined predictors of CPM that occurred between the date on which test results were disclosed and the time of CPM election. For patients who had not elected CPM by the time of data analysis, times were censored at the last contact at which the patient was known not to have elected CPM, alive, or dead. The median follow‐up time was 8.6 months. The distribution of time to CPM election for each group was estimated using the Kaplan–Meier method 22. The log‐rank test was used to determine the differences in the distributions of time to CPM between groups 23.

Regression analyses of time to CPM were conducted using the Cox proportional hazards model 24. Martingale residuals were used to check the function form of continuous variables, including age at diagnosis and at genetic testing, in the Cox proportional hazards models. A multivariable Cox proportional hazards model was obtained by first including a set of candidate predictor variables with a *P* value <0.10 in a univariate analysis. Because age at diagnosis and age at genetic testing were correlated, we chose age at genetic testing for the multivariable analysis because its association with time to CPM was more pertinent to answering our research question. Backward elimination was then performed using *P* < 0.05 for the significance level of the Wald chi‐square for an effect to stay in the model. Once the list of variables in our final model was selected, we further assessed the interaction effect on time to CPM between gene status and other variables to determine whether the predictors of electing CPM after genetic testing differed by final gene status. SAS version 9.4 and S‐Plus version 8.2 were used to carry out the computations for all analyses.

## Results

Table 1 shows patient demographic and clinical characteristics. Of the 314 patients, 70 elected CPM and 244 did not. Election of CPM by gene status was as follows: 30 of the 71 *BRCA1/BRCA2* carriers (42.3%), 22 of the 73 non‐*BRCA* carriers (30.1%), and 18 of the 170 with a VUS (10.6%). The mean age at genetic testing was 49.3 years.

**Table 1:** Demographic and clinical characteristics of patients with breast cancer who underwent multigene panel testing (*n* = 314)

Characteristic	No. (%)
Contralateral prophylactic mastectomy	
Yes	70 (22.3)
No	244 (77.7)
Personal history of ovarian cancer	
Yes	55 (17.5)
No	259 (82.5)
Marital status	
Divorced	29 (9.2)
Married	212 (67.5)
Separated	5 (1.6)
Single	39 (12.4)
Widowed	11 (3.5)
Unknown	18 (5.7)
Race/ethnicity	
Hispanic	46 (14.6)
White	203 (64.6)
Black	34 (10.8)
Other	29 (9.2)
Unknown	2 (0.6)
Education	
Advanced degree	45 (14.33)
Bachelor's degree	71 (22.61)
High school	11 (3.50)
Some college or technical school	14 (4.46)
Unknown	173 (55.10)
First‐degree family history of breast cancer	
0	215 (68.5)
≥1	97 (30.9)
Unknown	2 (0.6)
Total no. of relatives with a breast cancer diagnosis	
0	101 (32.2)
≥1	211 (67.2)
Unknown	2 (0.6)
First‐degree family history of ovarian cancer	
0	297 (94.6)
≥1	15 (4.8)
Unknown	2 (0.6)
Total no. of relatives with an ovarian cancer diagnosis	
0	262 (83.4)
≥1	50 (15.9)
Unknown	2 (0.6)
Estrogen receptor status	
Negative	69 (22.0)
Positive	223 (71.0)
Unknown	22 (7.0)
Progesterone receptor status	
Negative	103 (32.8)
Positive	187 (59.6)
Unknown	24 (7.6)
Nuclear grade	
I	29 (9.2)
II	112 (35.7)
III	136 (43.3)
Unknown	37 (11.8)
TNM stage	
0	31 (9.9)
1	89 (28.3)
2	106 (33.8)
3	53 (16.9)
4	18 (5.7)
Unknown	17 (5.4)
Gene status	
*BRCA* carrier	71 (22.6)
*BRCA* variant of uncertain significance	38 (12.1)
Non‐*BRCA* carrier	73 (23.2)
Non‐*BRCA* variant of uncertain significance	132 (42.0)
Hormone receptor status	
Positive	223 (71.0)
Negative	69 (22.0)
Unknown	22 (7.0)
Her2 status	
Positive	42 (13.4)
Negative	216 (68.8)
Unknown	56 (17.8)

Univariate analysis (Table 2) showed that patients aged 50 years or younger at the time of genetic testing were more likely to elect CPM (*P* = 0.0006). Educational status was also significantly associated with CPM such that those with advanced degree were likely to elect CPM than those lower educational level (*P* = 0.03).

**Table 2:** Univariate analysis of time to election of contralateral prophylactic mastectomy in patients with breast cancer who underwent multigene panel testing (*n* = 314)

Parameter	*P*	Hazard ratio	95% confidence interval	*P* for overall effect
Age at diagnosis				
≤50 years vs. >50	0.0399	1.919	1.031–3.574	0.0399
Age at genetic testing				
≤50 years vs. >50	0.0006	2.674	1.528–4.680	0.0006
Race/ethnicity				
Black vs. White	0.7602	0.890	0.420–1.883	0.6736
Hispanic vs. White	0.5465	0.811	0.410–1.604	
Others vs. White	0.2546	0.553	0.199–1.533	
Education				
Bachelor's degree/some college or technical school vs. advanced degree	0.0171	2.728	1.196–6.224	0.03
High school vs. advanced degree	0.7432	0.704	0.086–5.738	
Marital status				
Single, divorced, separated, or widowed vs. married	0.8376	1.057	0.621–1.801	0.8376
First‐degree family history of breast cancer yes vs. no	0.9782	0.993	0.599–1.645	0.9782
First‐degree family history of ovarian cancer yes vs. no	0.6614	0.772	0.243–2.456	0.6614
Personal history of ovarian cancer yes vs. no	0.0873	0.542	0.269–1.094	0.0873
Total no. of relatives with a breast cancer diagnosis ≥1 vs. 0	0.3914	1.255	0.747–2.108	0.3914
Total no. of relatives with an ovarian cancer diagnosis ≥1 vs. 0	0.7702	0.908	0.477–1.731	0.7702
Gene status				
*BRCA* carrier vs. VUS	<0.0001	4.695	2.611–8.441	<0.0001
Non‐*BRCA* carrier vs. VUS	<0.0001	3.689	1.973–6.896	
*BRCA* carrier vs. non‐*BRCA* carrier	0.3908	1.2727	0.7337–2.2076	
Nuclear grade				
III vs. I	0.3120	1.556	0.660–3.665	0.0560
II vs. I	0.6465	0.805	0.320–2.030	
Hormone receptor status				
Negative vs. positive	0.6237	1.144	0.668–1.960	0.6237
Her2 status				
Positive vs. negative	0.3755	1.345	0.698–2.593	0.3755
Lymphovascular invasion present vs. absent	0.8540	0.941	0.493–1.795	0.8540
Stage				
4 vs. 0	0.9518	0.958	0.239–3.838	0.2290
3 vs. 0	0.4653	0.666	0.224–1.983	
2 vs. 0	0.2638	1.643	0.688–3.928	
1 vs. 0	0.5213	1.348	0.541–3.361	

After adjustment for age, the multivariable Cox proportional hazards regression model revealed that *BRCA* carriers and non‐*BRCA* carriers were both more likely to elect CPM than those who had a VUS (*P* < 0.0001; Table 3). CPM election rates did not significantly differ between patients who were non‐*BRCA* carriers and those who were *BRCA* carriers (*P* = 0.6205; Table 3), even after adjustment for age.

**Table 3:** Multivariate Cox PH model of time to election of contralateral prophylactic mastectomy in patients with breast cancer who underwent multigene panel testing (*n* = 314)

Parameter		Hazard ratio	95% Hazard ratio confidence limits	*P*	*P* for overall effect
Gene Status	*BRCA* carriers vs. VUS	4.162	2.307–7.509	<.0001	<.0001
	Non‐*BRCA* carriers vs. VUS	3.618	1.932–6.775	<.0001	<.0001
	BRCA carriers vs. non‐*BRCA* carriers	1.1504	0.6606–2.004	0.6205	<.0001
Age at genetic testing	<50 vs. ≥50	2.321	1.321–4.080	0.0034	<.0001

**Figure 1:**
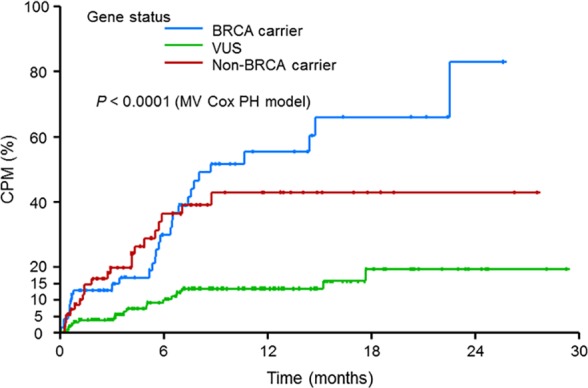
Cumulative incidence plot of contralateral prophylactic mastectomy among all patients.

A cumulative incidence plot for election of CPM after genetic testing showed that the rate of CPM election among *BRCA* carriers over 6 months and non‐*BRCA* carriers over 12 months was greater than the rate of CPM election among those who had a VUS for these periods (Table 4, Figure 1). More specifically, the rates of CPM election were as follows: among *BRCA* carriers, 29.9% over 6 months and 55.4% over 12 months; among non‐*BRCA* carriers, 36.4% over 6 months and 43% over 12 months; and among those who had a VUS, 9.2% over 6 months and 13.4% over 12 months (Table 4, Figure 1).

**Table 4:** Rates of contralateral prophylactic mastectomy (CPM) election by age at genetic testing and test results among patients with breast cancer who underwent multigene panel testing (*n* = 314)

Variable	CPM/total	CPM rate (range), %	
		6 months after genetic testing	12 months after genetic testing
Age at genetic testing			
<50 years	54/182	26.0 (19.3–34.4)	40.3 (31.8–50.0)
≥50 years	16/132	11.7 (6.9–19.6)	15.0 (9.1–24.3)
Genetic test results			
*BRCA* carrier	30/71	29.9 (19.5–44.2)	55.4 (41.1–70.8)
Variant of uncertain significance	18/170	9.2 (5.3–15.7)	13.4 (8.3–21.2)
Non‐*BRCA* carrier	22/73	36.4 (24.7–51.4)	43 (29.7–59.1)

Analysis of predictors of CPM election revealed an interaction between gene mutation status and hormone receptor status in predicting CPM election, after adjustment for age at genetic testing (*P* = 0.0053). As shown in Table 5, for *BRCA* carriers, hormone receptor status was not associated with CPM election (*P* = 0.5657), but for non‐*BRCA* carriers, negative hormone receptor status was associated with CPM election (*P* = 0.0115). For those with a VUS, hormone receptor status was not associated with CPM election (*P* = 0.1879). However, age ≤50 years was associated with CPM election in this group (*P* = 0.0286).

**Table 5:** Predictors of contralateral prophylactic mastectomy election among patients with breast cancer who underwent multigene panel testing (*n* = 314)

Variable	*P*	Hazard ratio	95% Confidence interval
Age <50 vs. ≥50 years			
*BRCA* carrier	0.5703	1.283	0.543–3.029
Non‐*BRCA* carrier	0.0941	2.382	0.862–6.578
Variant of uncertain significance	0.0286	3.801	1.150–12.56
Negative hormone receptor status			
*BRCA* carrier	0.5657	0.796	0.366–1.732
Non‐*BRCA* carrier	0.0115	3.278	1.305–8.230
Variant of uncertain significance	0.1879	0.316	0.057–1.756

## Discussion

Our results showed that, overall, 23% of patients with breast cancer who underwent multigene panel testing with a non‐negative result, including *BRCA* carriers, non‐*BRCA* carriers, and those with a VUS, opted for CPM, up from the average of 7% among breast cancer patients 20. Patients who had a pathogenic variant identified were more likely to elect CPM than those with a VUS, no matter which gene was implicated.

Moreover, patients aged 50 years or younger were more likely to elect CPM than older patients for all genes included in this cohort, and patients with an advanced educational level were also more likely to elect CPM that those with a lower educational level. Predictors and rates of CPM have already been established among patients carrying *BRCA1* or *BRCA2* pathogenic variants, but data are lacking regarding predictors and rates of CPM among patients who are carriers of non‐*BRCA* pathogenic variants or those with a VUS. Our study revealed that while the rate of CPM between *BRCA* carriers and non‐*BRCA* carriers was not significantly different, the predictors of CPM were different in each group.

Few studies have examined the rate and predictors of CPM among non‐*BRCA* carriers, although one noteworthy study by Kurian et al. 25 showed a similar trend of self‐reported CPM election to that of our study. Their study revealed that patients with a pathogenic variant in *BRCA1*/*BRCA2* or another gene associated with breast cancer were more likely to elect CPM than those with negative results or a VUS. Our study took this a step further to determine that there is no significant difference in CPM rate between *BRCA* carriers and non‐*BRCA* carriers. While these groups are receiving CPM at similar rates, it is important to note that their surgical recommendations and risk profiles are significantly different due to the lack of data on CBC rates in non‐*BRCA* carriers. Despite the lack of data on CBC risk and the absence of consensus guidelines recommending CPM, both studies confirm a trend of more invasive surgical intervention for individuals with breast cancer and any hereditary predisposition to cancer.

The rate of CPM election among *BRCA* carriers has been well established given that bilateral prophylactic mastectomy (BPM) reduces the risk of developing breast cancer by more than 90% 26. Among *BRCA* carriers, 65% of those with breast cancer and 15–60% of unaffected individuals elect prophylactic surgeries 27, 28, 29, 30. Factors associated with prophylactic surgery among *BRCA* carriers with breast cancer include age, type of initial breast cancer surgery, prophylactic oophorectomy, desire to have children, and family history of breast cancer 31, 32, 33, 34. Moreover, a study of CPM election rates among patients with ductal carcinoma in situ who were *BRCA*‐positive, *BRCA*‐negative, or untested showed that 27% opted for the surgery, and age, family history of ovarian cancer, and *BRCA* positivity predicted CPM election. The present study showed similar factors associated with CPM election in the cohort as a whole, including *BRCA* carriers, non‐*BRCA* carriers, and those with a VUS. The predictors varied, though, when comparing each of these groups with one another.

Among non‐*BRCA* carriers, negative hormone receptor status predicted CPM election. Moreover, previous studies without an emphasis on genetic testing results indicated that the risk of developing CBC in hormone receptor‐negative breast cancers is 1.6‐fold higher than in hormone receptor‐positive breast cancers 35. Given the increased risk, it stands to reason that patients with hormone receptor‐negative breast cancer are more likely to elect CPM than those with hormone receptor‐positive breast cancer 36. Additionally, Brewster et al. 37 found that CPM was associated with improved disease‐free survival in patients with hormone receptor‐negative breast cancer. Although these factors could explain the CPM election rate in the general population, their application to our population is less clear because genetic results were not included in that analysis. Future studies are needed to determine whether the same factors are evident among patients who undergo multigene panel testing. It is important to note that the small sample size in the group of patients who were non‐BRCA carries makes it hard to reach a solid conclusion that hormone receptor‐negative status is a significant factor for CPM among non‐BRCA carries. However, we think the magnitude of the hazard ratio associated with it is not negligible. This finding warrants further validation in future large studies. Additionally, the insignificant association between hormone receptor status and CPM for the BRCA carrier's group in this study could be due to the small sample size. This finding also needs to be further explored with a larger sample size.

Finally, age ≤50 years predicted CPM election for those who had a VUS in our analysis. Age is a well‐established significant predictor of CPM election among *BRCA* carriers 27, 28, 29, 30. Our study provided evidence that age also seems to affect the decision to elect CPM among those with a VUS. However, future studies with a larger sample are needed to confirm these findings further clarify how the VUS population differs from the general population of patients with breast cancer.

The decision‐making process for the election of CPM is complex and entails several driving forces, including the possible anxiety of receiving a positive genetic testing result. Studies suggest that CPM election may also be influenced by biopsies, screening costs and fatigue, cosmetic considerations, psychological factors, and perceived emotional advantages of CPM, among others 38, 39. Also, these patients had to make their decision about CPM on the basis of the treatment planned for their unilateral breast cancer, so their desire to avoid repeated treatment (CBC) could have influenced the decision.

Future prospective studies are needed to determine whether the same psychosocial factors influence the surgical decision making of individuals with hereditary cancer predispositions beyond *BRCA1* and *BRCA2*.

Despite the rapid growth of next‐generation sequencing allowing for simultaneous testing of multiple genes linked to cancer risk, the clinical impact of pathogenic variants in genes beyond *BRCA1* and *BRCA2*, especially in terms of CBC risk, is still largely unknown. Therefore, physicians are faced with the dilemma of making surgical recommendations without CBC risk data for genes often included on multigene panel tests marketed for patients with breast cancer. Our study indicates that patients are still opting for CPM as a measure of prevention even though substantive data on the potential need for this surgery is unavailable. It is also important to note that there are potential harms associated with CPM, such as postsurgical complications and concerns with body image, femininity, and sexuality 39. It is not clear that increased CPM in non‐*BRCA* carriers will improve disease‐specific or overall survival. While long‐term prospective studies are needed to determine whether CPM is the ideal clinical intervention for non‐*BRCA* carriers, our data suggest the need for additional provider and patient education on the known and unknown CBC risks to aid in treatment planning.

To the best of our knowledge, our study is the first to uniquely evaluate CPM election rates and predictors of CPM election among patients who are non‐*BRCA* carriers and those with a VUS. Our findings suggest that predictors of CPM election may differ among non‐*BRCA* carriers and those with a VUS from those who are *BRCA* carriers. Our findings also highlight the similarity in CPM election rates among *BRCA* carriers and non‐*BRCA* carriers despite the lack of evidence concerning CBC risk among those with a non‐*BRCA* mutation. This finding suggests that patients with a non‐*BRCA* mutation may have similar perception of CBC risk as those with a *BRCA* mutation. The follow‐up in this study time was less than a year from the time of genetic testing to date of CPM; it is important to highlight that the rate of CPM may increase with longer follow‐up. Future studies are needed to track CPM rate in this cohort for longer follow‐up period.

A few limitations of this study are worthy of consideration when interpreting the results. The authors recognize that is this a retrospective review in a cohort of patients who were referred for genetic counseling and testing at a large academic research hospital with genetic services integrated into the patient care team. Therefore, this population likely has a different level of post‐test education on their genetic testing results than the average breast cancer patient.

The predictors of CPM and issues of patient education may differ in hospital settings without easy access to genetic counseling. Our findings may need to be replicated in a study with a larger sample size to allow for generalizability; subgroup analyses should be interpreted with caution given the small sample sizes. Moreover, previous research has found an association between preoperative MRI and CPM 40. However, we did not collect information on the use of preoperative MRI in this cohort which posed another limitation to our findings. Future prospective studies need to address this association among individuals undergoing multigene panel testing. Finally, future prospective studies are needed to evaluate the complex decision‐making processes and the potential implication of genetic test results leading to CPM for individuals who undergo multigene panel testing. The present study identified demographic and clinical predictors of CPM in individuals undergoing multigene panel testing, but further research into patient's motivation to undergo CPM is needed.

## Conclusion

The present study reports CPM election rates specifically among patients with breast cancer who underwent multigene panel testing. Our analyses shed the light on the increasing use of CPM among patients who are non‐*BRCA* carriers as well those with a VUS. Our study elucidates the differing predictive factors of CPM election among *BRCA* carriers, non‐*BRCA* carries, and those with a VUS. These differing predictors may need to be considered in clinical recommendations for potential preventive surgeries. Our findings reveal the need for providers to be cognizant that non‐*BRCA* genes and VUS drive women to elect CPM despite the lack of data for CBC risk associated with these genes. Factors driving their decision need to be carefully addressed.

## Conflict of Interest

The manuscript has never been published and is not under consideration for publication elsewhere. The authors have no financial disclosures to declare.

## References

[1] Newman, B. , M. A. Austin , M. Lee , and M. C. King . 1988 Inheritance of human breast cancer: evidence for autosomal dominant transmission in high‐risk families. Proc. Natl Acad. Sci. USA 85:3044–3048. 3362861 10.1073/pnas.85.9.3044 PMC280139

[2] Claus, E. B. , N. Risch , and W. D. Thompson . 1991 Genetic analysis of breast cancer in the cancer and steroid hormone study. Am. J. Hum. Genet. 48:232–242. 1990835 PMC1683001

[3] Claus, E. B. , J. M. Schildkraut , W. D. Thompson , and N. J. Risch . 1996 The genetic attributable risk of breast and ovarian cancer. Cancer 77:2318–2324. 8635102 10.1002/(SICI)1097-0142(19960601)77:11<2318::AID-CNCR21>3.0.CO;2-Z

[4] Ford, D. , D. F. Easton , M. Stratton , S. Narod , D. Goldgar , P. Devilee , et al. 1998 Genetic heterogeneity and penetrance analysis of the BRCA1 and BRCA2 genes in breast cancer families. Am. J. Human Genet. 62:676–689. 9497246 10.1086/301749 PMC1376944

[5] Chen, S. , E. S. Iversen , T. Friebel , D. Finkelstein , B. L. Weber , A. Eisen , et al. 2006 Characterization of BRCA1 and BRCA2 mutations in a large United States sample. J. Clin. Oncol. 24:863–871. 16484695 10.1200/JCO.2005.03.6772 PMC2323978

[6] Heymann, S. , S. Delaloge , A. Rahal , O. Caron , T. Frebourg , L. Barreau , et al. 2010 Radio‐induced malignancies after breast cancer postoperative radiotherapy in patients with Li‐Fraumeni syndrome. Radiat. Oncol. 5:104. 21059199 10.1186/1748-717X-5-104 PMC2988810

[7] Hearle, N. , V. Schumacher , F. H. Menko , S. Olschwang , L. A. Boardman , J. J. Gille , et al. 2006 Frequency and spectrum of cancers in the Peutz‐Jeghers syndrome. Clin. Cancer Res. 12:3209–3215. 16707622 10.1158/1078-0432.CCR-06-0083

[8] Ngeow, J. , K. Stanuch , J. L. Mester , J. S. Barnholtz‐Sloan , and C. Eng . 2014 Second malignant neoplasms in patients with cowden syndrome with underlying Germline PTEN mutations. J. Clin. Oncol. 32:1818–1899. 24778394 10.1200/JCO.2013.53.6656 PMC4039869

[9] Pharoah, P. D. P. , P. Guilford , C. Caldas , and Int Gastric Canc Linkage C . 2001 Incidence of gastric cancer and breast cancer in CDH1 (E‐cadherin) mutation carriers from hereditary diffuse gastric cancer families. Gastroenterology 121:1348–1353. 11729114 10.1053/gast.2001.29611

[10] Tung, N. , S. M. Domchek , Z. Stadler , K. L. Nathanson , F. Couch , J. E. Garber , et al. 2016 Counselling framework for moderate‐penetrance cancer‐susceptibility mutations. Nat. Rev. Clin. Oncol. 13:581–588. 27296296 10.1038/nrclinonc.2016.90 PMC5513673

[11] Couch, F. J. , H. Shimelis , C. Hu , S. N. Hart , E. C. Polley , J. Na , et al. 2017 Associations between cancer predisposition testing panel genes and breast cancer. JAMA Oncol. 3:1190–1196. 28418444 10.1001/jamaoncol.2017.0424 PMC5599323

[12] Kriege, M. , A. Hollestelle , A. Jager , P. E. A. Huijts , E. M. Berns , A. M. Sieuwerts , et al. 2014 Survival and contralateral breast cancer in CHEK2 1100delC breast cancer patients: impact of adjuvant chemotherapy. Br. J. Cancer 111:1004–1013. 24918820 10.1038/bjc.2014.306 PMC4150261

[13] Mellemkjaer, L. , C. Dahl , J. H. Olsen , L. Bertelsen , P. Guldberg , J. Christensen , et al. 2008 Risk for contralateral breast cancer among carriers of the CHEK2*1100delC mutation in the WECARE Study. Br. J. Cancer 98:728–733. 18253122 10.1038/sj.bjc.6604228 PMC2259175

[14] Broeks, A. , L. de Witte , A. Nooijen , A. Huseinovic , J. G. Klijn , F. E. van Leeuwen , et al. 2004 Excess risk for contralateral breast cancer in CHEK2*1100delC germline mutation carriers. Breast Cancer Res. Treat. 83:91–93. 14997059 10.1023/B:BREA.0000010697.49896.03

[15] De Bock, G. H. , M. Schutte , E. M. M. Krol‐Warmerdam , C. Seynaeve , J. Blom , C. T. M. Brekelmans , et al. 2004 Tumour characteristics and prognosis of breast cancer patients carrying the germline CHEK2*1100delC variant. J. Med. Genet. 41:731–735. 15466005 10.1136/jmg.2004.019737 PMC1735606

[16] Bernstein, J. L. , R. W. Haile , M. Stovall , J. D. Jr Boice , R. E. Shore , B. Langholz , et al. 2010 Radiation exposure, the ATM gene, and Contralateral breast cancer in the women's environmental cancer and radiation epidemiology study. J. Natl Cancer Inst. 102:475–483. 20305132 10.1093/jnci/djq055 PMC2902825

[17] Broeks, A. , L. M. Braaf , A. Huseinovic , M. K. Schmidt , N. S. Russell , F. E. van Leeuwen , et al. 2008 The spectrum of ATM missense variants and their contribution to contralateral breast cancer. Breast Cancer Res. Treat. 107:243–248. 17393301 10.1007/s10549-007-9543-6 PMC2137941

[18] Concannon, P. , R. W. Haile , A. L. Børresen‐Dale , B. S. Rosenstein , R. A. Gatti , S. N. Teraoka , et al. 2008 Variants in the ATM gene associated with a reduced risk of contralateral breast cancer. Cancer Res. 68:6486–6491. 18701470 10.1158/0008-5472.CAN-08-0134 PMC2562548

[19] Tischkowitz, M. , M. Capanu , N. Sabbaghian , L. Li , X. Liang , M. P. Vallée , et al. 2012 Rare germline mutations in PALB2 and breast cancer risk: a population‐based study. Hum. Mutat. 33:674–680. 22241545 10.1002/humu.22022 PMC3767757

[20] Wong, S. M. , R. A. Freedman , Y. Sagara , F. Aydogan , W. T. Barry , and M. Golshan . 2017 Growing use of contralateral prophylactic mastectomy despite no improvement in long‐term survival for invasive breast cancer. Ann. Surg. 265:581–589. 28169929 10.1097/SLA.0000000000001698

[21] Angelos, P. , I. Bedrosian , D. M. Euhus , V. M. Herrmann , S. J. Katz , and A. Pusic . 2015 Prophylactic mastectomy: challenging considerations for the surgeon. Ann. Surg. Oncol. 22:3208–3212. 26259752 10.1245/s10434-015-4758-y PMC4836440

[22] Kaplan, E. L. 1958 Non‐parametric estimation from incomplete observation. Am. J. Stat. Assoc. 53:457–458.

[23] Mantel, N. 1966 Evaluation of survival data and two new rank order statistics arising in its consideration. Cancer Chemother. Rep. 50:163–170. 5910392

[24] Cox, D. R. 1972 Regression models and life‐tables. J. R. Stat. Soc. B‐Stat. Methodol. 34:187–220.

[25] Kurian, A. W. , Y. Li , A. S. Hamilton , K. C. Ward , S. T. Hawley , M. Morrow , et al. 2017 Gaps in incorporating germline genetic testing into treatment decision‐making for early‐stage breast cancer. J. Clin. Oncol. 35:2232–2239. 28402748 10.1200/JCO.2016.71.6480 PMC5501363

[26] Kurian, A. W. , Y. Li , A. S. Hamilton , K. C. Ward , S. T. Hawley , M. Morrow , et al. 2004 Bilateral prophylactic mastectomy reduces breast cancer risk in BRCA1 and BRCA2 mutation carriers: the PROSE study group. J. Clin. Oncol. 22:1055–1062. 14981104 10.1200/JCO.2004.04.188

[27] Chung, A. , K. Huynh , C. Lawrence , M.‐S. Sim , and A. Giuliano . 2012 Comparison of patient characteristics and outcomes of contralateral prophylactic mastectomy and unilateral total mastectomy in breast cancer patients. Ann. Surg. Oncol. 19:2600–2606. 22396004 10.1245/s10434-012-2299-1

[28] Stuckey, A. , D. Dizon , J. S. Wilbur , J. Kent , T. Tejada‐Berges , J. Gass , et al. 2010 Clinical characteristics and choices regarding risk‐reducing surgery in BRCA mutation carriers. Gynecol. Obstet. Invest. 69:270–273. 20090358 10.1159/000276573

[29] Wainberg, S. , and J. Husted . 2004 Utilization of screening and preventive surgery among unaffected carriers of a BRCA1 or BRCA2 gene mutation. Cancer Epidemiol. Biomark. Prev. 13:1989–1995. 15598752

[30] Skytte, A. B. , A. M. Gerdes , M. K. Andersen , L. Sunde , K. Brøndum‐Nielsen , M. Waldstrøm , et al. 2010 Risk‐reducing mastectomy and salpingo‐oophorectomy in unaffected BRCA mutation carriers: uptake and timing. Clin. Genet. 77:342–349. 20059483 10.1111/j.1399-0004.2009.01329.x

[31] Heemskerk‐Gerritsen, B. A. , C. T. Brekelmans , M. B. Menke‐Pluymers , A. N. van Geel , M. M. Tilanus‐Linthorst , C. C. Bartels , et al. 2007 Prophylactic mastectomy in BRCA1/2 mutation carriers and women at risk of hereditary breast cancer: long‐term experiences at the rotterdam family cancer clinic. Ann. Surg. Oncol. 14:3335–3344. 17541692 10.1245/s10434-007-9449-x PMC2077910

[32] Litton, J. K. , S. N. Westin , K. Ready , C. C. Sun , S. K. Peterson , F. Meric‐Bernstam , et al. 2009 Perception of screening and risk reduction surgeries in patients tested for a BRCA deleterious mutation. Cancer 115:1598–1604. 19280625 10.1002/cncr.24199 PMC2680417

[33] Meijers‐Heijboer, H. , B. van Geel , W. L. van Putten , S. C. Henzen‐Logmans , C. Seynaeve , M. B. Menke‐Pluymers , et al. 2001 Breast cancer after prophylactic bilateral mastectomy in women with a BRCA1 or BRCA2 mutation. N. Engl. J. Med. 345:159–164. 11463009 10.1056/NEJM200107193450301

[34] Metcalfe, K. A. , A. Finch , A. Poll , D. Horsman , C. Kim‐Sing , J. Scott , et al. 2009 Breast cancer risks in women with a family history of breast or ovarian cancer who have tested negative for a BRCA1 or BRCA2 mutation. Br. J. Cancer 100:421–425. 19088722 10.1038/sj.bjc.6604830 PMC2634722

[35] Brown, D. , S. Shao , I. Jatoi , C. D. Shriver , and K. M. Zhu . 2016 Trends in use of contralateral prophylactic mastectomy by racial/ethnic group and ER/PR status among patients with breast cancer: a SEER population‐based study. Cancer Epidemiol. 42:24–31. 26999400 10.1016/j.canep.2016.02.011

[36] Jones, N. B. , J. Wilson , L. Kotur , J. Stephens , W. B. Farrar , and D. M. Agnese . 2009 Contralateral prophylactic mastectomy for unilateral breast cancer: an Increasing Trend at a Single Institution. Ann. Surg. Oncol. 16:2691–2696. 19506956 10.1245/s10434-009-0547-9

[37] Brewster, A. M. , I. Bedrosian , P. A. Parker , W. Dong , S. K. Peterson , S. B. Cantor , et al. 2012 Association between contralateral prophylactic mastectomy and breast cancer outcomes by hormone receptor status. Cancer 118:5637–5643. 22517269 10.1002/cncr.27574 PMC3478501

[38] Hoskins, L. M. , and M. H. Greene . 2012 Anticipatory loss and early mastectomy for young female BRCA1/2 mutation carriers. Qual. Health Res. 22:1633–1646. 22927701 10.1177/1049732312458182

[39] Tesson, S. , I. Richards , D. Porter , K. A. Phillips , N. Rankin , D. Costa , et al. 2017 Women's preferences for contralateral prophylactic mastectomy following unilateral breast cancer: what risk‐reduction makes it worthwhile? Breast 31(Suppl. C):233–240. 27969575 10.1016/j.breast.2016.11.025

[40] Xia, C. , M. C. Schroeder , R. J. Weigel , S. L. Sugg , and A. Thomas . 2014 Rate of contralateral prophylactic mastectomy is influenced by preoperative MRI recommendations. Ann. Surg. Oncol. 21:4133–4138. 24934585 10.1245/s10434-014-3852-x PMC4255269

